# Mechanistic deconvolution of BSA size variants by constrained Raman pseudo‐Voigt hard modeling during anion‐exchange chromatography

**DOI:** 10.1002/btpr.88534

**Published:** 2026-06-25

**Authors:** Jakob Heyer‐Müller, Robin Schiemer, Lars Robbel, Michael Schmitt, Jürgen Hubbuch

**Affiliations:** ^1^ Institute of Process Engineering in Life Sciences—Section IV: Biomolecular Separation Engineering Karlsruhe Institute of Technology (KIT) Karlsruhe Baden‐Württemberg Germany; ^2^ CSL Innovation GmbH Germany Marburg Hessen Germany

**Keywords:** aggregation, chemometrics, chromatography, process analytical technology, Raman spectroscopy, spectral hard modeling

## Abstract

In biopharmaceutical manufacturing, protein aggregation is a critical quality attribute, necessitating rapid and reliable analytical strategies during downstream processes like anion‐exchange chromatography (AEX). While Raman spectroscopy enables continuous monitoring of protein secondary structure, standard data‐driven regression models struggle to decouple intrinsic structural changes from gradient‐induced solvent and buffer drifts under dynamic chromatographic conditions. Addressing this methodological gap, this study establishes a constrained pseudo‐Voigt hard modeling framework for the mechanistic deconvolution of bovine serum albumin (BSA) size variants during in‐line Raman monitoring of AEX processes. By explicitly defining a parametric background model to capture salt‐induced spectral drift, the methodology effectively isolates matrix variations from genuine protein‐specific signals. The constrained hard model was applied to 285 in‐line spectra across diverse chromatographic conditions, achieving reconstruction fidelity while maintaining stable, physically interpretable component identities. The mechanistically derived Amide I center of mass emerged as a robust, aggregation‐sensitive descriptor that preserves structural information despite strong concentration dynamics. Furthermore, the extracted spectral features demonstrated strong predictive performance for monomer concentration and acceptable accuracy for high molecular weight components. Collectively, these results demonstrate that constrained spectral hard modeling provides a highly interpretable, robust, and calibration‐light alternative to classical partial least squares approaches for the real‐time monitoring of protein size variants.

## 1 INTRODUCTION

Protein aggregation is a critical quality attribute in biopharmaceutical manufacturing, necessitating robust analytical strategies for rapid detection during downstream separation processes like anion‐exchange chromatography (AEX).^1^, ^2^, ^3^ While Raman spectroscopy enables continuous monitoring of protein secondary structure via the structurally sensitive Amide I vibrational band, standard data‐driven methods, such as partial least squares (PLS) regression, struggle under dynamic chromatographic conditions,^4^, ^5^, ^6^, ^7^, ^8^ protein structure assessment,^9^ reaction monitoring.^10^ Gradient elution introduces pronounced solvent and buffer‐related spectral drifts that PLS cannot reliably decouple from intrinsic structural changes, resulting in entangled, calibration‐heavy models that lack physical interpretability. To circumvent these limitations, spectral hard modeling integrates mechanistic prior knowledge by representing overlapping bands as a linear superposition of defined mathematical functions (e.g., pseudo‐Voigt profiles).^11^, ^12^, ^13^ By enforcing physically motivated constraints, hard modeling preserves component identity and isolates background interference from genuine analyte variance. However, robustly resolving the highly overlapping contributions of solvent, monomeric proteins, and aggregated species within the narrow Amide I window remains a fundamental challenge for dynamic bioprocesses.^14^, ^15^, ^16^, ^17^, ^18^ Addressing this methodological gap requires a systematic evaluation of spectral deconvolution under realistic operational conditions, guided by three core questions: How can mechanistic prior knowledge effectively isolate gradient‐induced matrix effects from protein‐specific structural signals? How accurately can individual spectral contributions corresponding to background, monomeric, and aggregated species be resolved within the dynamic Amide I envelope? To what extent do mechanistically derived spectral features improve physical interpretability without sacrificing the predictive performance required for quantitative process monitoring? To answer these questions, this work establishes a constrained pseudo‐Voigt hard modeling framework for the mechanistic deconvolution of bovine serum albumin (BSA) size variants during in‐line Raman monitoring of AEX processes. By explicitly defining a parametric background model to capture salt‐induced spectral drift, this methodology isolates solvent variations and extracts physically interpretable parameters for the robust, real‐time resolution of protein monomers and aggregates.

## 2 MATERIALS AND METHODS

### 2.1 Experimental

#### 2.1.1 Bind–elute AEX

Bind–elute AEX chromatography experiments with in‐line Raman spectroscopy were conducted to generate process data for the separation of BSA monomers and aggregates under process‐relevant conditions with dynamically changing buffer composition. Experiments were performed using a 5 mL Eshmuno Q column (Merck KGaA, Darmstadt, Germany) on an Äkta Pure system (Cytiva, Uppsala, Sweden). Unstressed BSA feed solutions with a total protein concentration of 15g/L were prepared using a single commercial lot of lyophilized powder (Sigma‐Aldrich, St. Louis, MO, USA). To evaluate the impact of feedstock preparation and reconstitution reproducibility, two independent batches were prepared separately by dissolving the identical starting material in 20mM Tris at pH 8. These separate dissolution procedures resulted in native baseline high molecular weight component (HMWC) contents of 25.33% for Batch 1 and 15.67% for Batch 2, as characterized via SE‐UPLC reference analytics.

It is important to emphasize that no artificial stress mechanisms (such as heat, pH excursions, or chemical denaturation) were applied to induce aggregation. The HMWC fraction represents the inherent baseline aggregation of the commercial lyophilized powder, consisting primarily of non‐covalently associated oligomers and native disulfide‐linked dimers formed during industrial manufacturing, lyophilization, and subsequent storage of the purchased material prior to reconstitution. Utilizing this unstressed material ensures that the Raman spectra reflect realistic, process‐relevant aggregation pathways rather than artificially denatured conformations. While an initial aggregate content of 15% to 25% is unusually high for a commercial‐grade protein, this elevated baseline is highly advantageous for the present study. The primary objective of this work is to rigorously evaluate the spectral hard modeling workflow and its mathematical decoupling capabilities across a broad dynamic range of size variants, making the absolute purity of the starting material secondary to its structural diversity.

Prior to sample loading, the column was equilibrated for 5 column volumes (CVs) using 20 mM Tris (pH 8). Following sample loading, the column was washed for 5 CVs with equilibration buffer to remove unbound components and stabilize the baseline prior to elution. Gradient elution was subsequently performed using linear NaCl gradients ranging from 0 to 500 mM NaCl at different gradient lengths. After the gradient phase, a strip step at 1 M NaCl was applied for 5 CVs to remove strongly retained species. The column was then regenerated using 1 M NaOH for 5 CVs and re‐equilibrated with equilibration buffer to restore the initial conditions. All elution phases were operated at a constant flow rate of 1 mL/min. During the elution phase, fractions were collected at intervals of 200 μL. Raman spectra were recorded in‐line at an exposure time of 500 ms and a laser power of 495 mW, resulting in 24 individual acquisitions per fraction. To preserve temporal resolution during the chromatographic process, single 500 ms acquisitions were used. These acquisitions were subsequently averaged over the time intervals corresponding to each ultrahigh‐performance size‐exclusion chromatography (UHP‐SEC)‐analyzed fraction to enable quantitative comparison with fraction‐wise analytics. The Raman measurement point was incorporated into the flow path between the outlet valve and the fractionator, ensuring that the recorded spectra correspond to the effluent immediately upstream of fractionation. In total, six AEX runs were performed using different gradient lengths, loading densities, and feedstocks derived from two independent BSA batches. Loading densities were intentionally scaled in direct proportion to the gradient lengths across the experiments. This experimental design was deployed to break any systematic, co‐linear mathematical correlation between absolute protein elution concentration profiles and local salt kinetics, thereby strictly validating the decoupling capability of the hard‐modeling framework. A summary of the experimental conditions, including the assignment to training and test sets, is provided in Table 1. Despite the variance in the absolute initial aggregate percentages induced by the separate reconstitution events, SE‐UPLC analytics confirmed that both prepared feedstocks exhibited a highly consistent distribution profile of dimeric and higher‐order oligomeric species. This structural consistency justifies the treatment of the HMWC fraction as a functionally homogeneous class during spectral feature extraction. Throughout this manuscript, these chromatographic datasets serve as the basis for evaluating the robustness of the hard modeling workflow.

**TABLE 1:** Experimental conditions for bind‐elute anion‐exchange chromatography (AEX) experiment used in in‐line Raman monitoring of bovine serum albumin (BSA) monomer and aggregate separation.

Run ID	Gradient length (CVs)	Load density (mg/mL resin)	Total feed concentration (mg/mL)	HMWC content (%)
Batch 1 5CV	5	15	15	25.33
Batch 1 10CV	10	17.5	15	25.33
Batch 1 15CV	15	22.5	15	25.33
Batch 2 5CV	5	22.5	15	15.67
Batch 2 10CV	10	30	15	15.67
Batch 2 15CV	15	45	15	15.67

#### 2.1.2 Raman acquisition settings

Raman spectroscopy measurements were performed using a Raman BioReactor BallProbe inserted into a Raman flow cell with a dead volume of 240 μL (both MarqMetrix, Seattle, WA, USA) connected to a HyperFlux™ PRO Plus 785 spectrometer operated using SpectralSoft 3.2.600.1 software (Tornado Spectral Systems, Mississauga, Ontario, Canada). All Raman measurements were performed at the maximum laser power of 495 mW. To preserve temporal resolution during continuous process measurements, the exposure time was set to 500 ms per acquisition.

#### 2.1.3 UHP‐SEC

To quantify the distribution of protein size variants and provide fraction‐wise reference analytics for the Raman measurements, samples were analyzed using UHP‐SEC. A TSKgel SuperSW mAb HTP column (4 μm particle size, 4.6×150mm) was operated at a flow rate of 0.3 mL/min. The mobile phase consisted of 15 mM sodium phosphate buffer at pH 6.2. Prior to injection, samples were diluted to protein concentrations within the range of 0–1 mg/mL to ensure operation within the linear response regime and to prevent column overloading. Analyses were performed using a Vanquish UHPLC system controlled by Chromeleon software (version 7.2) (Thermo Fisher Scientific, Waltham, MA, USA). For each fraction, the absolute concentrations of monomeric and aggregated protein species were determined by peak integration. These concentrations were subsequently used for (i) qualitative comparison with Raman‐derived spectral markers and (ii) quantitative evaluation of model‐derived trajectories.

### 2.2 Data analysis

All data analysis and computations were performed in Python 3.12.7. The analysis workflow consisted of multiple steps, including (i) spectral preprocessing, (ii) definition and fitting of a constrained background model for gradient‐induced matrix effects, (iii) constrained pseudo‐Voigt hard modeling of the protein Amide I band, (iv) extraction of physically interpretable parameters and derived spectral markers, and (v) quantitative evaluation of model‐derived trajectories. All processing steps were applied consistently across chromatographic runs to ensure comparability of the fitted parameters and marker trajectories.

#### 2.2.1 Spectral preprocessing

Raman spectral processing consisted of multiple steps, including truncation, normalization, background subtraction, baseline correction, smoothing, feature selection, and derivative computation. Each operation was chosen to suppress non‐chemical variability while retaining aggregation‐relevant structural information in the Amide I region.

##### Truncation

All spectra were first truncated to 500–3250 cm^−1^. The lower bound of 500 cm^−1^ was chosen as smaller wavenumbers solely contain baseline contributions and bands stemming from the sapphire window built into the probe head. The upper bound of 3250 cm^−1^ corresponds to the maximum of the water stretching band within the accessible spectral range and was retained to ensure consistent normalization.^19^

##### Normalization

Spectra were subsequently normalized using index normalization to the local baseline minimum adjacent to the C—H stretching region at 2750 cm^−1^. While the general methodological concept of utilizing the C—H stretching envelope as a stable scaling reference under aqueous, protein‐containing conditions is derived from Dietrich et al.,^20^ the specific wavenumber of 2750 cm^−1^ was selected empirically in this study. This frequency is established as a reliable intensity anchor point due to the absence of intrinsic protein or salt vibrational modes in the immediate region preceding the aliphatic C—H stretching profile.^21^ Index‐based normalization was preferred over global area normalization to avoid confounding changes in broadband intensity contributions from water bands[cite: 75]. Unlike standard normal variate (SNV) normalization, which scales based on global spectral variance and can distort peak ratios when solvent backgrounds change dynamically, index‐based scaling to this local baseline anchor remains unaffected by salt‐induced drifts in water intensity.

##### Background subtraction

Subsequently, background subtraction was performed to reduce solvent‐ and buffer‐related contributions prior to baseline correction. A representative protein‐free buffer spectrum originating from the start of the respective gradient elution phase, where no protein signal was detectable, was selected for each chromatographic experiment. This buffer spectrum was subtracted from all subsequently acquired spectra on an index‐wise basis. Background subtraction was performed to improve visualization of protein‐related spectral features and stabilize downstream baseline correction, particularly in the Amide I region, where water and buffer contributions can overlap with protein vibrations. As the NaCl concentration changes continuously during gradient elution, background subtraction alone is insufficient to fully remove salt‐induced spectral variation. Residual salt‐dependent changes were therefore explicitly addressed in the hard modeling framework by parametric background modeling (Section 3.1.1).

##### Baseline correction

Furthermore, baseline correction was performed using a Whittaker smoothing approach through the derivative peak‐screening asymmetric least squares algorithm (DERPSALSA) as implemented in the *pybaselines* library (v. 1.1.0), with parameters $\lambda =10^{5}$, k=0.02, and d=2.^22^ These parameters were selected to suppress broad baseline components (e.g., fluorescence, instrument drift, residual scattering) while preserving peak shapes and relative intensities in the Amide I window.

##### Smoothing and derivatives

Finally, high‐frequency noise reduction was performed using a Savitzky–Golay (SG) filter as implemented in *scipy* (v. 1.14.1), with a second‐order polynomial and a window size of 21 points. Smoothed spectra were used for visualization and as inputs for peak initialization. The SG filter was also employed for second‐derivative computation using the same filter configuration to enhance subtle spectral variations and support robust peak localization in overlapping band regions (Figure 1). The second derivative was explicitly preferred over the first derivative as it simultaneously eliminates linear baseline slopes and converts convoluted inflection shoulders into distinct, sharp downward minima. In the present work, derivative spectra were used exclusively for peak localization and initialization; all final fitting and quantitative marker extraction were performed on the corresponding smoothed (non‐derivative) spectra to preserve physically meaningful intensity information.

**FIGURE 1:**
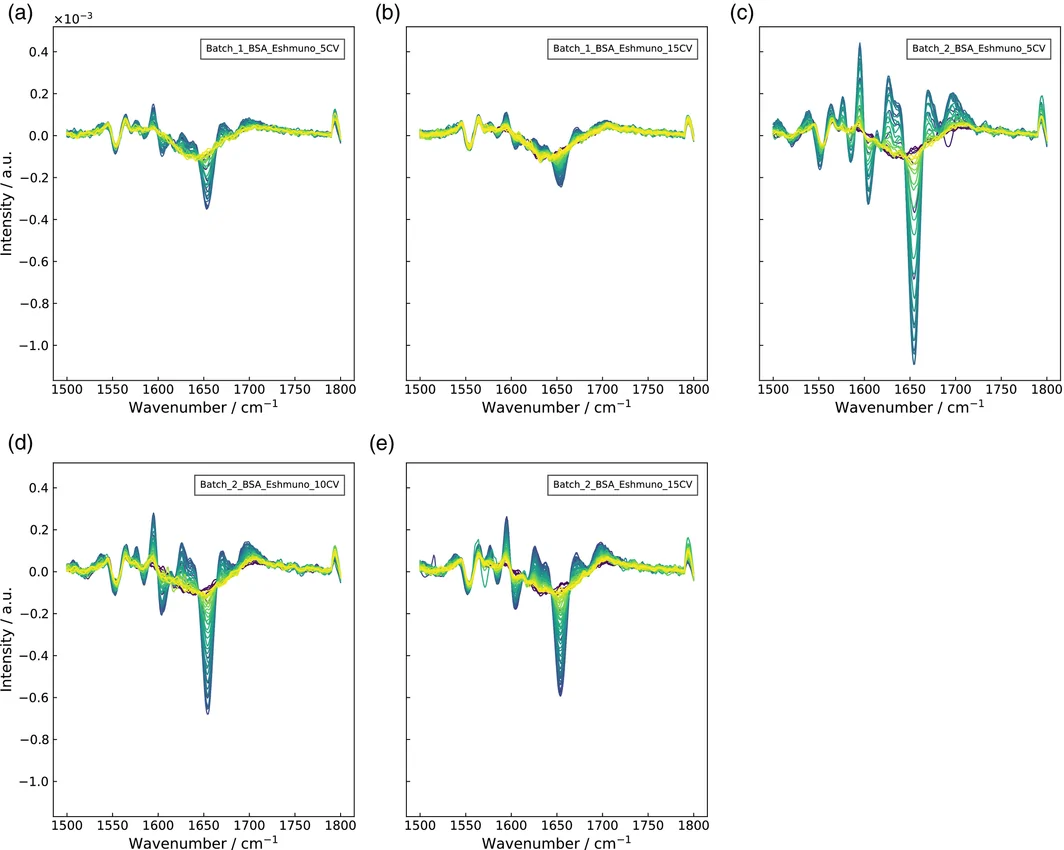
Comparison of second‐derivative Raman spectra in the Amide I region across all chromatographic experiments. Subplots show the spectral variations corresponding to all chromatographic runs in a wavenumber range of 1500 to 1800 cm^−1^.

##### Feature selection

All downstream analyses were restricted to the Amide I region (1500–1801 cm^−1^), which is sensitive to protein secondary structure and aggregation‐related conformational changes. Restricting the spectral window was performed to reduce the number of fitted degrees of freedom, improve the numerical stability of constrained fitting, and focus the hard model on the most structurally informative region. For the remainder of this manuscript, “Amide I” refers to this window.

## 3 RESULTS AND DISCUSSION

### 3.1 Hard modeling framework

To enable a mechanistic and physically interpretable decomposition of Amide I Raman spectra into contributions from solvent/buffer and protein structure, a hard modeling framework was developed. In contrast to purely data‐driven multivariate approaches, the hard model enforces component identity across spectra by constraining peak centers and widths, enabling direct comparison of fitted parameters between fractions and experiments. The hard model was designed to disentangle (i) intrinsic structural changes associated with aggregation from (ii) extrinsic matrix‐induced spectral variation under gradient elution, thereby supporting robust analysis of size variant behavior under process conditions.

#### 3.1.1 Buffer background modeling and salt‐induced effects

During bind–elute AEX chromatography, gradient elution induces systematic changes in buffer composition, primarily through increasing NaCl concentrations. These changes affect the Raman spectra through solvent‐related contributions, including the H—O—H bending vibration and additional oxygen‐related bands overlapping with the Amide I region.^4^ To prevent these effects from being absorbed into protein‐associated components during spectral deconvolution, background contributions were explicitly modeled as a low‐dimensional parametric hard model prior to protein analysis. A protein‐free buffer reference spectrum was obtained from the first spectrum of the 5 CV gradient elution segment, assuming negligible protein contribution. To ensure numerical stability and consistent parameterization, the reference spectrum was interpolated to a uniform wavenumber grid ranging from 1500 to 1800 cm^−1^ with a spacing of 1 cm^−1^. This interpolation ensures that peak localization and pseudo‐Voigt evaluation operate on an identical spectral grid across all chromatographic runs. Candidate background peak positions were subsequently identified using a continuous wavelet transform (CWT)^23^ with a Mexican hat (Ricker) wavelet.^24^ Wavelet coefficients were computed across multiple scales and summed over low‐to‐intermediate scales to emphasize peak‐like structures in the presence of residual noise. The resulting peak candidates were refined by restricting the search to the range 1550–1700 cm^−1^, thereby focusing on solvent‐related contributions overlapping with the Amide I region while excluding regions dominated by protein bands. The background contribution in the Amide I region was represented as the superposition of two pseudo‐Voigt components (visualized in Figure 2a),

(1) $$y_{\mathrm{bg}}\left(\nu \right)=\sum_{k=1}^{2}A_{k}^{\mathrm{bg}}\cdot \mathrm{PV}\left(\nu ;\nu_{k}^{\mathrm{bg}},\sigma_{k}^{\mathrm{bg}},\eta_{k}^{\mathrm{bg}}\right),$$

with initial center estimates located near 1550 and 1640 cm^−1^. Model parameters were estimated using nonlinear least‐squares optimization and stored as a reference background model for subsequent analysis. To capture the dominant solvent‐ and buffer‐related features within the Amide I window while avoiding overparameterization, the two‐component model was selected as a parsimonious representation.

**FIGURE 2:**
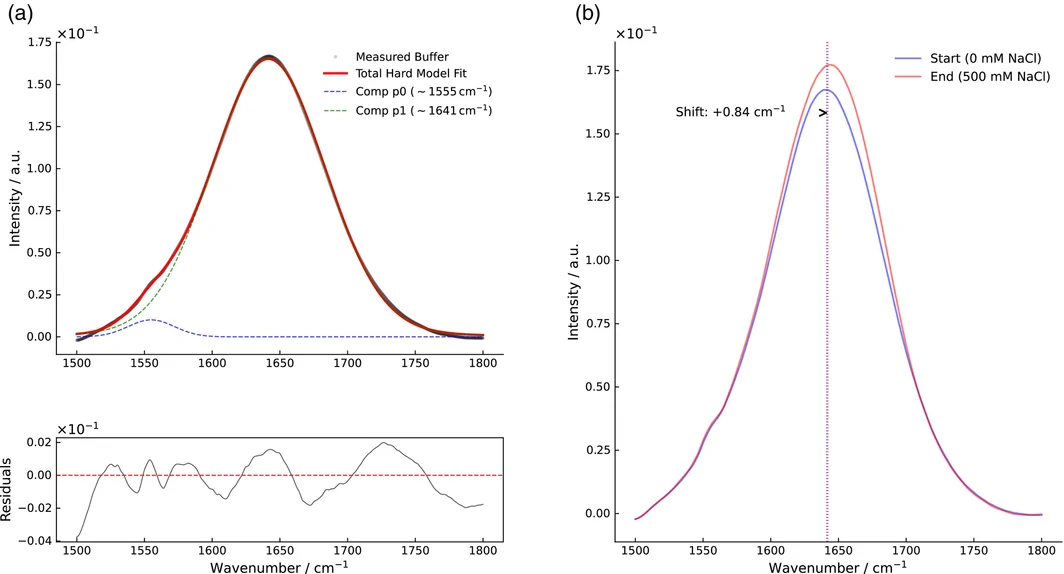
Background modeling and salt‐induced spectral drift during gradient elution in the Amide I region. (a) Measured buffer spectrum overlaid with total hard model fit, demonstrating underlying components p0 (1555 cm^−1^) and p1 (1641 cm^−1^), alongside fitting residuals. (b) Spectral drift occurring between start (0 mM NaCl) and the end condition (500 mM NaCl).

To quantify gradual salt‐induced spectral changes during gradient elution, the reference background model was fitted to buffer spectra acquired across the NaCl gradient using three constraint strategies. First, a global scaling approach was applied in which the background model was multiplied by a non‐negative constant factor c≥0 calculated independently per spectrum to dynamically compensate for frame‐wise linear fluctuations in absolute baseline intensity caused by path length variations while keeping all pseudo‐Voigt parameters fixed. Second, a fully flexible refit was evaluated that allowed all background parameters ($A_{k}^{\mathrm{bg}}$, $\nu_{k}^{\mathrm{bg}}$, $\sigma_{k}^{\mathrm{bg}}$, $\eta_{k}^{\mathrm{bg}}$) to vary independently for each spectrum. Third, a partially flexible refit was implemented in which the model was kept fixed except for selected parameters of the second background component, namely the center position and amplitude, which were allowed to vary to capture salt‐driven spectral shifts and intensity changes while maintaining model parsimony (Figure 2b). The resulting parameter trajectories served two purposes: (i) providing empirical evidence of the magnitude and direction of salt‐driven drift in the Amide I background and (ii) defining the constrained background treatment employed during protein hard modeling to prevent confounding between background and protein components.

A dedicated parametric background model was fitted to protein‐free buffer spectra and subsequently utilized as a constrained background contribution during protein deconvolution within the Amide I window (1500–1800 cm^−1^). The reference background fit yielded excellent agreement with the measured buffer spectrum, with a coefficient of determination of $R^{2}=0.99954$ and an root mean squared error (RMSE) of $1.225\times 10^{-3}$ in model intensity units, confirming that the selected low‐dimensional pseudo‐Voigt representation adequately captures the dominant solvent‐related contributions in this spectral region. The reference background model comprised two pseudo‐Voigt components centered at approximately 1555 and 1641.5 cm^−1^. These peak positions are consistent with solvent‐dominated contributions overlapping the Amide I region and align with the expected spectral signatures of water‐related bending modes and associated background features under aqueous buffer conditions. To assess salt‐induced drift during gradient elution, the background model was fitted to the first and last buffer spectra of each elution experiment. Across all experiments, a systematic drift of the dominant water/background component (p1) was observed. The center position exhibited shifts of $\Delta \nu_{p1}=+0.12$ to +0.89 cm^−1^, while the corresponding amplitude increased by $\Delta A_{p1}=+0.18$ to +1.55 in model intensity units. Despite these gradual changes, the background‐only fits remained highly stable throughout the elution, with $R_{\text{start}}^{2}=0.99934–0.99959$ at the beginning and $R_{\mathrm{end}}^{2}=0.99932–0.99961$ at the end of the gradient. These results demonstrate that gradient elution introduces systematic matrix drift even in protein‐free spectra, confirming that explicit background treatment is required for mechanistic interpretation in the Amide I region. By isolating solvent‐ and buffer‐related variation in dedicated components (p0–p1), the protein‐associated components can be fitted under stable constraints without absorbing gradient‐driven covariance. This approach directly addresses a known limitation of purely data‐driven models, which often entangle concentration effects and matrix variation and therefore require extensive recalibration when process conditions change.

#### 3.1.2 Model definition

The measured Amide I spectrum $y\left(\nu \right)$ was modeled as a linear superposition of pseudo‐Voigt components and a residual term,

(2) $$y\left(\nu \right)=\sum_{i=1}^{N}A_{i}\cdot \mathrm{PV}\left(\nu ;\nu_{i},\sigma_{i},\eta_{i}\right)+\varepsilon \left(\nu \right),$$

where $A_{i}$ denotes the component amplitude, $\nu_{i}$ the center position, $\sigma_{i}$ the width parameter, and $\eta_{i}$ the Lorentzian fraction of component i. The pseudo‐Voigt function was selected to flexibly capture intermediate peak shapes between the Gaussian and Lorentzian limits while maintaining interpretable parameters. Solvent‐related contributions were addressed by the dedicated background model and were not absorbed into the protein component parameters during protein deconvolution.

#### 3.1.3 Parameter initialization and constraints

Constrained optimization necessitates stable initialization and physically motivated bounds to ensure consistent component identity across spectra and to prevent non‐physical solutions. Initial peak center estimates were systematically and automatically obtained from a Mexican‐hat wavelet analysis applied to smoothed second‐derivative spectra, thereby eliminating manual trial‐and‐error initialization. The Mexican hat wavelet was selected due to its sensitivity to localized curvature changes and its analytical relationship to the derivatives of Gaussian functions, which enables robust peak localization in overlapping band regions. The wavelet‐derived centers were used exclusively as initialization points and to define narrow center boundaries; wavelet‐transformed spectra were not subjected to fitting or quantitative interpretation. Peak center positions $\nu_{i}$ were constrained within component‐specific bounds aligned with known vibrational assignments and empirically observed peak neighborhoods in the Amide I region. These constraints prevent peak swapping and enforce consistent component identity across fractions and experiments, which is essential for interpreting parameter trajectories as mechanistic indicators. All amplitudes were constrained to non‐negative values ($A_{i}\geq 0$) to enforce physically meaningful contributions. No upper bounds were imposed, allowing the amplitudes to reflect concentration‐driven changes during chromatographic elution. The width parameters $\sigma_{i}$ were constrained to physically plausible ranges to prevent overfitting of noise through artificially narrow peaks or compensatory broadening that could mask the underlying spectral structure. These width constraints were kept constant across all spectra to maintain comparability of the fitted components and to limit the effective degrees of freedom.

#### 3.1.4 Optimization strategy

Parameter estimation was performed hierarchically to decouple solvent‐driven background variability from protein‐related contributions and to stabilize the spectral deconvolution under process conditions. First, a parametric background model was established as described in Section 3.1.1. For protein‐containing spectra, the background parameters were not jointly optimized with the protein parameters. Instead, the background contribution was applied as a fixed or partially constrained term depending on the selected salt‐effect strategy, thereby preventing matrix‐driven variance from being absorbed into the protein components. Subsequently, spectra from early elution fractions containing exclusively monomeric protein species were used to calibrate a reference protein model (cf. Figure 3a). During this step, the background contribution was accounted for using the background model, while the protein peak centers and widths were optimized and subsequently fixed. Only the amplitudes of the protein components were allowed to vary across spectra. This calibration establishes a reference monomer state that anchors the component interpretation and minimizes parameter drift across the chromatographic run. For spectra containing aggregated species, the calibrated monomer parameters were retained to preserve structural consistency and ensure that amplitude changes remain interpretable as concentration effects. Additional pseudo‐Voigt components associated with aggregation‐related conformational changes were introduced while maintaining the constrained background treatment (cf. Figure 3b). This approach separates concentration‐driven amplitude variation from aggregation‐specific changes in band shape and shoulder contributions. Finally, all model parameters were estimated on a per‐spectrum basis using constrained nonlinear least‐squares optimization. Bounds and fixed‐parameter settings were enforced consistently across all spectra to ensure that the fitted parameters and derived markers remain comparable across time, fractions, and experiments. This strategy reduces parameter cross‐correlation and limits compensatory solutions between the background and protein components.

**FIGURE 3:**
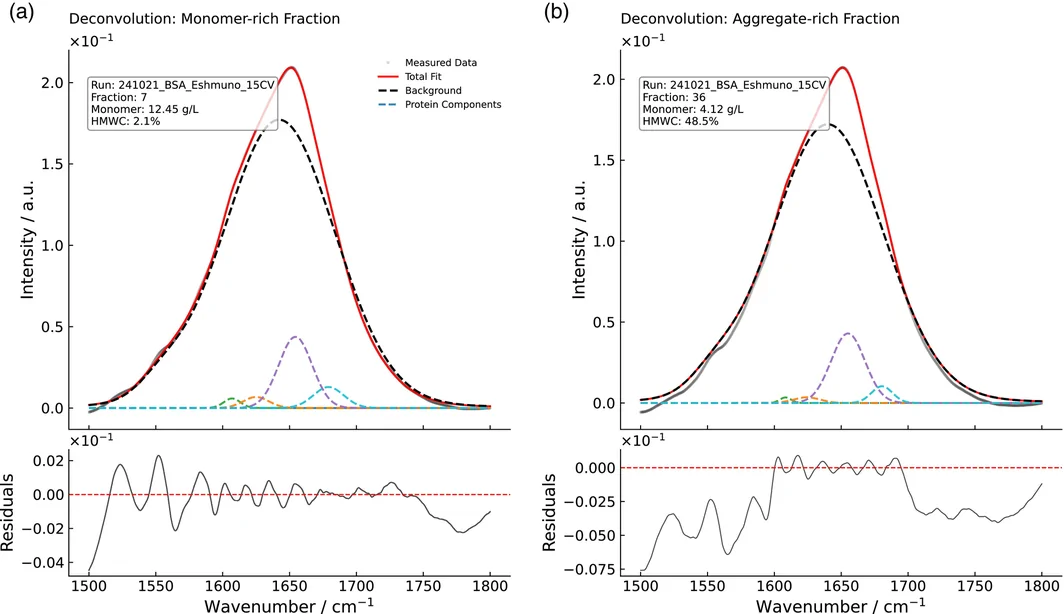
Representative constrained pseudo‐Voigt deconvolution of Amide I spectra. (a) Deconvolution of a monomer‐rich fraction yielding 12.45 g/L monomer and 2.1% high molecular weight component (HMWC). (b) Deconvolution of an aggregate‐rich fraction yielding 4.12 g/L monomer and 48.5% HMWC. Both panels plot the measured data, total fit, background, individual protein components, and model residuals.

#### 3.1.5 Model validation and predictive evaluation

Model performance was evaluated using complementary criteria that assess both numerical fit quality and the predictive relevance of the extracted hard‐model parameters. First, the quality of the spectral fits was examined using several diagnostic metrics. The coefficient of determination ($R^{2}$) was used as the primary measure of explained variance within the fitted spectral window, while residual spectra were inspected to identify systematic deviations such as unmodeled shoulders or baseline curvature that would indicate insufficient model complexity or inappropriate parameter constraints. In addition, reduced $\chi^{2}$ values were used to evaluate fit quality relative to the expected noise level and the number of fitted parameters, providing a complementary criterion to $R^{2}$ for detecting potential overfitting or underfitting. Reduced $\chi^{2}$ values close to unity indicate that the residuals are consistent with the expected noise magnitude. To balance fit accuracy and model parsimony, model complexity was further evaluated using the Akaike information criterion (AIC). The AIC was used to compare candidate model configurations and to ensure that additional components were justified by improved explanatory power rather than by overparameterization. Because AEX elution introduces pronounced concentration dynamics across the chromatographic profile, these fit diagnostics were evaluated across the entire elution trajectory, including low‐signal front and tail fractions. This approach provides a realistic estimate of model behavior under process analytical technology (PAT)‐relevant conditions, where early detection of co‐elution or tailing effects can be more critical than the accurate fitting of high‐intensity apex fractions. Beyond spectral fit quality, the predictive relevance of the parameters extracted from the hard modeling framework was evaluated using a grouped cross‐validation strategy. This analysis aimed to determine whether hard‐model‐derived features contain transferable information regarding protein concentration and aggregation‐related critical quality attributes across independent chromatographic runs. For this purpose, experiment‐wise hard‐model outputs were loaded from stored result dictionaries containing the fitted parameter tables, residual spectra, and reconstructed Amide I signals. Derived spectral descriptors, including the Amide I center of mass and the 1654/1610 cm^−1^ intensity ratio, were used together with the amplitudes of all fitted pseudo‐Voigt components as candidate predictor variables. Corresponding reference values for monomer, aggregate, and total protein concentrations were obtained from the associated result structures to compute the aggregate fraction. Samples with total protein concentrations below 0.01 g/L were excluded from the regression analysis to ensure a meaningful protein signal contribution. Predictive performance was assessed using a leave‐one‐experiment‐out cross‐validation scheme. This grouping strategy prevents overly optimistic performance estimates that could arise from randomly splitting highly correlated neighboring fractions originating from the same chromatographic run. Consequently, the resulting performance reflects the ability of hard‐model‐derived features to generalize to previously unseen experiments. Within each cross‐validation fold, the selected features were standardized using a z‐score transformation fitted exclusively on the training data. Regression models were trained using ridge regression with internal selection of the regularization strength, providing a transparent linear baseline capable of handling correlated predictor variables while reducing coefficient instability. The evaluated feature set consisted of the amplitudes of all fitted pseudo‐Voigt components and the Amide I center of mass. Separate regression models were calibrated for the prediction of monomer concentration, aggregate concentration, and aggregate fraction. Model performance was quantified for each fold using the coefficient of determination and the root‐mean‐square error on the corresponding test set. This evaluation provides a run‐wise estimate of how effectively mechanistically derived hard‐model parameters support quantitative prediction of protein size variants under previously unseen chromatographic conditions.

### 3.2 Dataset coverage and hard‐model execution

The constrained hard modeling workflow was applied to all in‐line Raman spectra acquired during the AEX elution phases and mapped to fraction‐wise UHP‐SEC labels. In total, 285 fraction‐averaged spectra were evaluated across six AEX experiments, including 5 CVs, 10 CVs, and 15 CVs linear gradients, as well as step elution experiments originating from two independent batches. As determined by UHP‐SEC reference analytics, the total protein concentration spanned a range of 0–79.40 mg/mL, thereby encompassing the full dynamic elution window from baseline fractions to peak maxima (Figure 4). The aggregate fraction ranged from 0 to 100%, with extreme values predominantly observed in low‐signal tail fractions. This broad coverage of concentration and aggregate concentrations ensures that model behavior is evaluated under realistic chromatographic conditions rather than only in apex fractions with favorable signal‐to‐noise ratios. Furthermore, the inclusion of low‐signal and tail fractions is particularly relevant for PAT applications, as these regions are often critical for pooling and process decisions. The hard model was structured as a superposition of constrained pseudo‐Voigt components within the Amide I window. Specifically, two components (p0–p1) were assigned to solvent and background contributions, while five components (p2–p6) were utilized to describe the protein‐associated Amide I envelope. This component structure was kept constant across all spectra, which is essential for maintaining parameter comparability and for interpreting amplitude changes mechanistically rather than as model reconfiguration artifacts.

**FIGURE 4:**
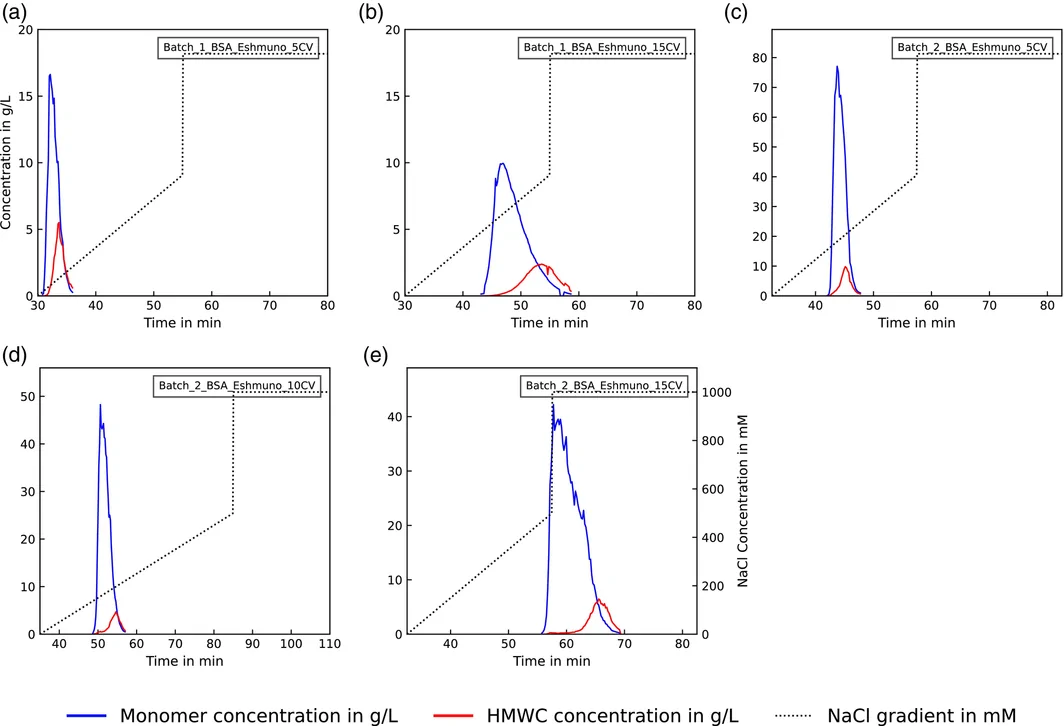
Comparison of monomer and high molecular weight component (HMWC) elution profiles across the investigated anion‐exchange chromatography (AEX) experiments. Blue traces represent monomer concentration, red traces represent HMWC concentration in g/L, and dotted black lines represent the NaCl gradient in mM. The *x*‐axis plots the dynamic window strictly from the initiation of the linear elution gradient to the completion of the column strip step.

### 3.3 Hard‐model spectral reconstruction quality

The constrained pseudo‐Voigt hard model captured the Amide I band shape with high fidelity across the complete chromatographic elution profiles. Across all evaluated spectra (n=285), a global goodness‐of‐fit of $R^{2}=0.99890\pm 0.00118$ (mean±SD) was achieved, spanning a range from $R_{\min}^{2}=0.99548$ to $R_{\max}^{2}=0.99975$. Within the modeled 1500–1800 cm^−1^ window, the corresponding global reconstruction error amounted to $\text{RMSE}=1.98\times 10^{-3}\pm 1.17\times 10^{-3}$. This uniformly high reconstruction fidelity demonstrates that the constrained hard model is sufficiently flexible to describe the Amide I envelope without requiring unconstrained overparameterization. Furthermore, a concentration‐dependent trend in fit stability was observed. The lowest goodness‐of‐fit values occurred in very low‐signal tail fractions, where the total protein concentration approached baseline levels and the Amide I contribution became comparable to residual noise. This slight decrease in fit quality in low‐signal fractions is expected and reflects signal‐to‐noise limitations rather than systematic model failure. Restricting the evaluation to fractions with increased total protein concentration reduced the influence of near‐baseline spectra and yielded consistently high fit quality across experiments, with experiment‐wise $R_{\min}^{2}$ values ranging from 0.9959 to 0.9996. Inspection of the residual spectra revealed no persistent band‐shaped deviations. Instead, the residuals were dominated by high‐frequency noise without systematic structure. This absence of structured residuals indicates that the selected number of components and the imposed constraints were sufficient to describe the Amide I envelope under dynamically changing gradient conditions, thereby capturing genuine spectral information rather than compensating for missing features through non‐physical parameter drift.

### 3.4 Resolved protein components and peak constraints

Across all experiments, the five protein‐associated pseudo‐Voigt components remained confined to narrow and physically plausible center ranges, thereby ensuring consistent component identity and comparability between fractions and batches. Upon pooling all spectra, the mean center positions and observed ranges were as follows: p2 at 1562.20 cm^−1^ (1559.00–1569.00 cm^−1^), p3 at 1605.16 cm^−1^ (1599.00–1609.00 cm^−1^), p4 at 1623.55 cm^−1^ (1615.67–1625.00 cm^−1^), p5 at 1654.05 cm^−1^ (1651.00–1661.00 cm^−1^), and p6 at 1679.51 cm^−1^ (1672.00–1682.00 cm^−1^). Such stable center positions across experiments are a central prerequisite for interpreting the fitted components as physically meaningful and comparable descriptors rather than purely numerical basis functions. Furthermore, no systematic peak swapping or boundary violations were observed across all chromatographic runs. For all spectra, the fitted center positions remained within the predefined constraint intervals, and inter‐experimental variability was limited to small shifts within these bounds. Consequently, the resulting parameter distributions were unimodal for each component, showing no evidence of bimodal behavior or experiment‐specific clustering (Figure 5). The absence of peak swapping or boundary accumulation confirms that the chosen initialization and constraints are sufficiently informative to stabilize the optimization. Together with the high fit quality, these findings support the use of amplitude trajectories and derived markers as mechanistically interpretable outputs of the hard model. Given the strictly linear nature of the NaCl gradients (0 to 500 mM NaCl), the elution buffer composition can be tracked along the adjusted time axis. Monomer elution occurred predominantly between 150and280mMNaCl, while the HMWC fraction enriched in the tail regions up to 400mMNaCl before entering the high‐salt column strip.

**FIGURE 5:**
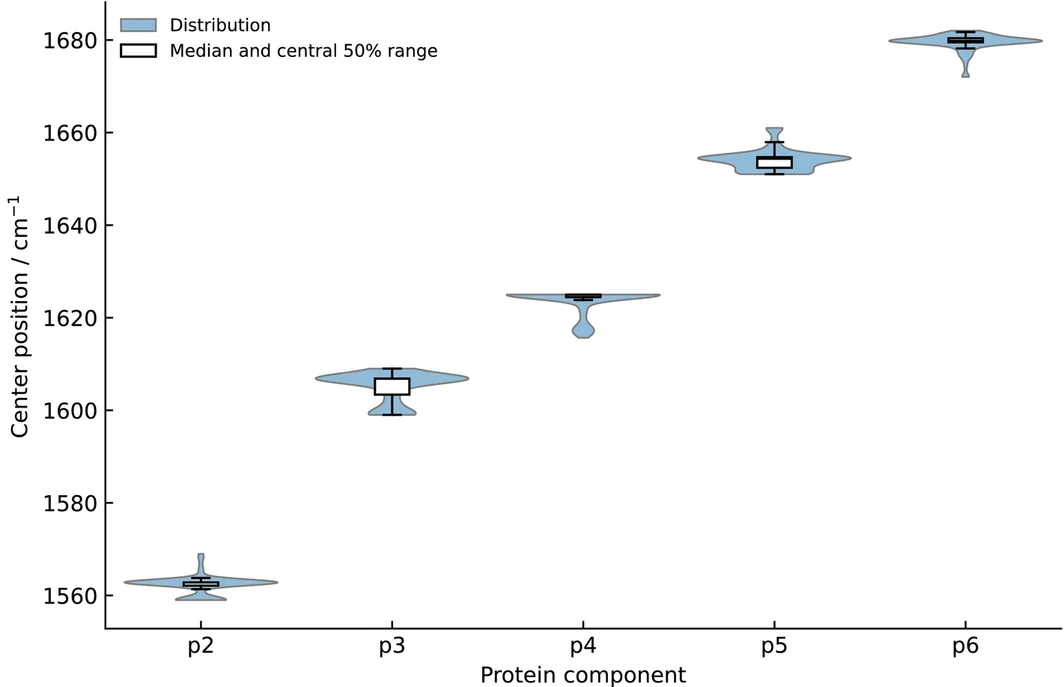
Distribution of fitted center positions (cm^−1^) for the protein‐associated pseudo‐Voigt components across all spectra. The violin plots (blue) depict the underlying probability distribution for components p2 through p6, the inner box plots denote the median and central 50% range.

### 3.5 Quantitative validation of hard‐model‐derived spectral features

To evaluate aggregation sensitivity across the full chromatographic concentration range, including low‐signal front fractions, apex fractions, and aggregate‐enriched tail fractions, spectral markers were extracted from the reconstructed protein‐only signal following the removal of background contributions. Across the complete dataset (n=285), aggregation‐dependent trends were detectable throughout the elution profile. However, marker variance increased in low‐signal fractions where absolute Raman intensities approached the detector noise level. In these regions, the Amide I contribution becomes comparable to residual noise, affecting the numerical stability of intensity‐based descriptors and increasing the dispersion of center‐of‐mass (CoM) estimates. Despite this increased variability, systematic aggregation‐related trends remained observable across the entire dataset.

Across all fractions, the CoM exhibited a positive monotonic relationship with increasing aggregate fraction. For fractions with $v_{\mathrm{agg}}<0.05$, the CoM amounted to 1643.32±2.31 cm^−1^ (n=85). In the intermediate aggregation range of $0.20\leq v_{\mathrm{agg}}<0.40$, the CoM increased to 1647.56±3.96 cm^−1^ (n=43). For higher aggregate fractions of $0.40\leq v_{\mathrm{agg}}<0.80$, the CoM reached 1651.29±5.45 cm^−1^ (n=49). These systematic shifts indicate that aggregation‐related structural changes within the Amide I envelope are captured by the hard‐model decomposition across the entire chromatographic concentration regime. Consequently, the CoM emerged as the most robust aggregation‐sensitive descriptor because it integrates the full Amide I envelope and is therefore less susceptible to local noise contributions than single‐point or ratio‐based markers.

In addition to the CoM, a ratiometric marker defined as $I_{1654}/I_{1610}$ was calculated from the reconstructed protein signal. Across the complete dataset, this ratio exhibited larger variance than the CoM due to numerical instability when the denominator intensity approached zero in low‐signal fractions. In particular, when the intensity at 1610 cm^−1^ approached the noise floor, inflated ratio values and heavy‐tailed distributions were observed. The instability of the $I_{1654}/I_{1610}$ ratio in low‐signal fractions highlights an important limitation of pointwise markers for online process decisions, especially when denominator intensities approach the noise floor. Nevertheless, the ratiometric marker also showed a positive association with aggregate fraction, increasing on average from 3.65 for $v_{\mathrm{agg}}<0.05$ to 8.40 for $0.40\leq v_{\mathrm{agg}}<0.80$. Compared to the CoM, the ratio remained more sensitive to noise contributions and component cross‐correlation effects, particularly in intermediate concentration fractions.

To assess whether the parameters extracted from the hard‐model decomposition contain predictive information regarding protein concentration and aggregation‐related attributes, the amplitudes of all fitted pseudo‐Voigt components together with the Amide I CoM were used as features in a multivariate regression analysis. Using a ridge regression model trained on the reconstructed spectral features, the apparent predictive performance across all gradient experiments yielded a coefficient of determination of $R^{2}=0.979$ for monomer concentration and $R^{2}=0.557$ for HMWC concentration (Figure 6). Absolute concentration units (g/L) are intentionally retained throughout the analysis to ensure mathematical stability across the dynamic elution profile. In front and tail fractions where total protein concentration approaches baseline levels, relative percentage metrics (%) become highly sensitive to minor noise floor fluctuations, whereas absolute mass concentration (g/L) provides an un‐skewed representation of the actual mass balance. The stronger apparent predictive performance for monomer concentration than for HMWC concentration is consistent with the dominance of the monomer contribution in the chromatographic dataset and the lower abundance of aggregate‐rich fractions. To contextualize these quantitative outcomes, a rigorous comparison with the current literature status quo for Raman‐based HMWC and protein purity tracking is warranted. The absolute cross‐validation test error of 1.587g/L achieved in this study translates to a relative error range of 2.0%–4.5% across the peak fractions, where total protein concentrations reach up to 79.40mg/mL. While conventional PLS models optimized for stable, matrix‐matched monoclonal antibody (mAb) formulations or steady‐state bioprocesses can achieve lower cross‐validation errors—with values frequently reported in narrow bounds such as 0.046%–0.801%,^25^ 0.037%–0.089%,^26^ 0.15%–0.52%,^7^ or 0.12%–0.65% ^8^—these purely data‐driven baselines are structurally constrained to unvarying chemical backgrounds. In such steady‐state literature studies, the solvent matrix does not undergo pronounced dynamic shifts that could mimic or confound genuine structural protein vibrations. In contrast, the presented hard‐modeling framework successfully handles massive gradient matrix variations across a 0to500mMNaCl elution window without requiring product‐specific, extensive calibration sets or large labeled training libraries.

**FIGURE 6:**
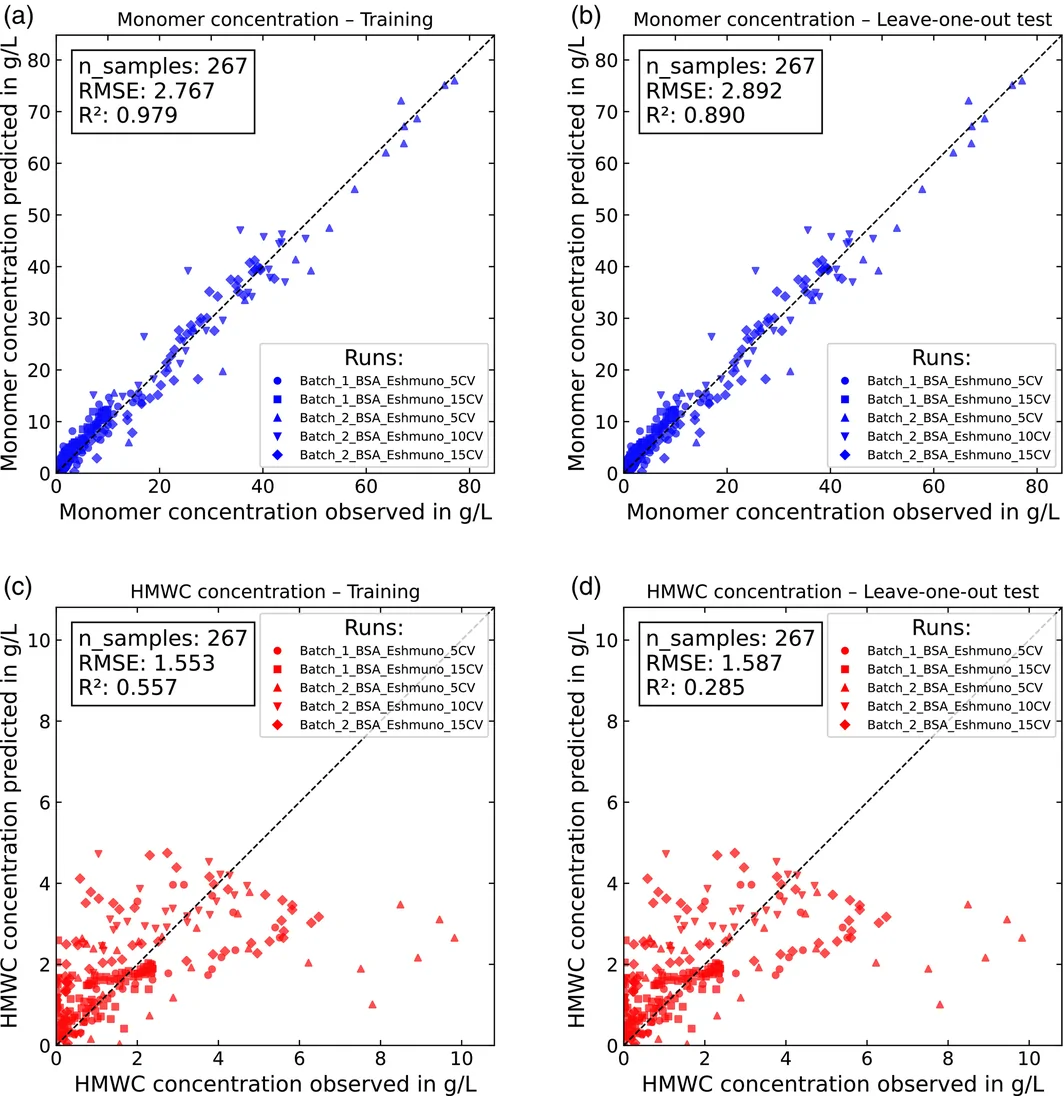
Model validation for monomer and high molecular weight component (HMWC) prediction based on hard‐model‐derived spectral features across *n* = 267 samples. (a) Monomer concentration prediction on the training set, (b) Monomer concentration prediction using a leave‐one‐out test set, (c) HMWC concentration prediction on the training set, (d) HMWC concentration prediction using a leave‐one‐out test set.

Consequently, the multivariate regression model for HMWC serves primarily as a qualitative screening tool, whereas precise tracking of aggregation kinetics is achieved via the more robust Center of Mass (CoM) descriptor.

A systematic under‐prediction of HMWC is observed primarily in the late tail fractions where total protein concentrations reach approximately 10g/L and the aggregate ratio equals or exceeds 50%. Because the baseline protein hard model was mathematically calibrated using early fractions containing purely monomeric species, rapid nonlinear multi‐component shape deformations within these extreme aggregate‐rich zones induce minor cross‐correlation errors between adjacent protein components (p4,p5,andp6).

Overall, the analysis reveals a concentration‐dependent hierarchy in marker robustness. The CoM represents a comparatively stable descriptor of aggregation‐related spectral changes across the chromatographic profile, while the ratiometric marker exhibits higher sensitivity to noise‐driven denominator fluctuations. Collectively, these findings indicate that hard‐model‐derived parameters consistently reflect aggregation‐induced spectral shifts within the reconstructed protein signal, demonstrating their capacity to serve both as mechanistically interpretable markers and as structured feature sets for downstream quantitative models. For practical bioprocess implementations, such as automated peak‐cutting operations, several caveats must be considered. Due to the high test uncertainty associated with the HMWC concentration regression model ($R_{\text{test}}^{2}=0.285$), direct utilization of the absolute predicted mass values (g/L) for fraction pooling decisions is not recommended. Instead, industrial implementation must rely on tracking the integrated Amide I Center of Mass (CoM) descriptor. This parameter operates as a robust, noise‐insensitive indicator of structural shape deformation. Fraction cutting should be executed by triggering pooling boundaries when the continuous CoM trajectory exceeds an empirical baseline threshold established during the pure monomer elution phase.

## 4 CONCLUSION

The implementation of the constrained pseudo‐Voigt hard modeling framework successfully integrates mechanistic prior knowledge, enabling a highly interpretable spectral decomposition that effectively separates solvent and background contributions from protein‐associated Amide I components during gradient elution. By relying on physically constrained spectral components rather than instrument‐specific latent variables, the workflow offers a robust, calibration‐light alternative to classical PLS models, thereby reducing the dependence on large labeled datasets and facilitating transferability across different process conditions. Furthermore, explicit background modeling accurately captured systematic salt‐induced spectral drift, ensuring that gradient‐driven matrix covariance was successfully isolated and prevented from being erroneously absorbed into the protein‐associated components. The mechanistically derived spectral features demonstrated exceptional stability and strong apparent predictive performance for monomer concentration ($R^{2}=0.979$), confirming that the structured feature sets capture the dominant variance of the dataset.

HMWC were captured with a training $R^{2}$ of 0.557 and a leave‐one‐out cross‐validation test $R^{2}$ of 0.285. While this validation score is low for strict quantitative concentration regression, the performance is evaluated as acceptable and passable exclusively from a PAT screening perspective, where continuous trend tracking is prioritized over absolute mass‐balance precision.

Finally, the Amide I center of mass, extracted from the reconstructed protein signal, emerged as a highly robust, aggregation‐sensitive descriptor that preserves structural information despite strong concentration dynamics. This circumvents the noise‐driven instabilities of pointwise markers and serves as a promising candidate for online pooling decisions. While the current study focuses on BSA as a model protein, the framework is methodologically transferable to other complex systems, such as monoclonal antibodies, by adjusting the initial peak constraints to the respective native secondary structure.

## AUTHOR CONTRIBUTIONS

**Jakob Heyer‐Müller:** Conceptualization; investigation; writing – original draft; methodology; validation; visualization; writing – review and editing; software; formal analysis; project administration; data curation. **Robin Schiemer:** Conceptualization; software; methodology. **Lars Robbel:** Resources. **Michael Schmitt:** Resources. **Jürgen Hubbuch:** Funding acquisition; project administration; supervision; resources.

## CONFLICT OF INTEREST STATEMENT

The authors declare no conflicts of interest.

## Data Availability

The data that support the findings of this study are available from the corresponding author upon reasonable request.
